# Auswirkungen des Ahrtal-Hochwassers auf die Gesundheit der lokalen Bevölkerung – eine Analyse auf Grundlage von GKV-Routinedaten

**DOI:** 10.1007/s00103-023-03809-x

**Published:** 2024-01-09

**Authors:** Jobst Augustin, Valerie Andrees, Alice Czerniejewski, Roman Dallner, Christian M. Schulz, Nikolaus Christian Simon Mezger

**Affiliations:** 1 Institut für Versorgungsforschung in der Dermatologie und bei Pflegeberufen (IVDP), Universitätsklinikum Hamburg-Eppendorf (UKE), Martinistraße 52, 20246 Hamburg, Deutschland; 2 BKK-Landesverband NORDWEST KdöR, Essen, Deutschland; 3 KLUG – Deutsche Allianz Klimawandel und Gesundheit e. V., Berlin, Deutschland; 4 Medizincampus Oberfranken, Institut für Medizinmanagement und Gesundheitswissenschaften, Universität Bayreuth, Bayreuth, Deutschland; 5 Centre for Planetary Health Policy, Berlin, Deutschland; 6 Global Public Health Department, Karolinska Institut, Stockholm, Schweden; 7 Arbeitsgruppe Globale und Planetare Gesundheit, Medizinische Fakultät, Martin-Luther-Universität Halle-Wittenberg, Halle (Saale), Deutschland

**Keywords:** Klimawandel, Extremwetterereignis, Flut, Erkrankung, Deutschland, Climate change, Extreme weather event, Flood, Disease, Germany

## Abstract

**Hintergrund:**

In den letzten Jahrzehnten traten in Deutschland Hochwasserereignisse auf, die eine Bedrohung für die Gesundheit der lokalen Bevölkerung darstellten. Es existieren allerdings kaum Studien, die die gesundheitlichen Folgen dieser Ereignisse untersuchen. Studienziel war daher die Untersuchung der Assoziationen des Ahrtal-Hochwassers im Jahr 2021 mit der Gesundheit der lokalen Bevölkerung.

**Methoden:**

Datengrundlage dieser Studie sind bundesweite Abrechnungsdaten (stationär/ambulant) des BKK-Landesverbands Nordwest. Untersuchungsregion war die Region Ahrweiler, Untersuchungszeiträume jeweils das 3. Quartal der Jahre 2020 und 2021. Unter anderem mit Prevalence-Rate-Ratio-Tests wurde auf Grundlage von ICD-10-Kodierungen untersucht, welche Diagnosen (stationär/ambulant) räumlich und zeitlich in Assoziation mit dem Hochwasserereignis standen.

**Ergebnisse:**

Die Ergebnisse zeigen im stationären Bereich eine deutliche Zunahme abgerechneter Leistungen in einigen Diagnosegruppen gegenüber dem Vorjahr. Verzeichnet wurden insbesondere Zunahmen bestimmter F‑Diagnosen (*psychische und Verhaltensstörungen*) und S‑Diagnosen (*Verletzungen*) sowie verschiedener Diagnoseschlüssel innerhalb der Z‑Kodierungen (*Faktoren, die den Gesundheitszustand beeinflussen und zur Inanspruchnahme des Gesundheitswesens führen*). Im ambulanten Sektor wurde in vielen Diagnosegruppen (F- und Z‑Diagnosen) eine Abnahme identifiziert.

**Diskussion:**

Die Ergebnisse der Studie deuten darauf hin, dass vor allem die mentale Gesundheit der lokalen Bevölkerung und die Gesundheitsversorgung insgesamt (sektorspezifische Inanspruchnahme) vom Hochwasser beeinträchtigt wurden. Da Hochwasserereignisse zukünftig häufiger und stärker werden können, müssen die Maßnahmen zum Schutz der Bevölkerung und Gesundheitsinfrastruktur entsprechend angepasst werden.

## Abstract (en)

**Background:**

In recent decades, Germany has experienced flood events that posed a threat to the health of the local population. However, there is a paucity of studies on the health consequences of these events. Therefore, the aim of the study was to investigate the health consequences of the Ahr Valley flood in 2021.

**Methods:**

The data basis of this longitudinal study are nationwide billing data (inpatient/outpatient) of the BKK-Landesverband Nordwest. The study region was Ahrweiler and the study periods were the third quarters of 2020 and 2021. Among other things, prevalence rate ratio tests were used to determine which diagnoses (inpatient/outpatient) were spatially and temporally associated with the flood event on the basis of ICD-10 coding.

**Results:**

The results show a significant increase in billed services for some diagnosis groups in the inpatient sector. In particular, there was an increase in F diagnoses (mental and behavioural disorders), S diagnoses (injuries) and various diagnosis codes within Z codes (factors influencing health status and leading to healthcare utilisation). In the outpatient sector, a decrease was observed in many diagnosis groups (F and Z diagnoses).

**Conclusion:**

The results of the study showed that the mental health of the local population was particularly affected by the floods. Healthcare was also affected. As floods are expected to become more frequent and severe in the future, measures to protect the population and health infrastructure need to be adapted accordingly.

## Einleitung

Die anthropogene Klimakrise geht mit einer steigenden Häufigkeit und Intensität von Hochwasserereignissen an Küsten und im Inland einher [1]. Nicht nur der globale Süden mit Millionen Geschädigten etwa durch die Flut in Pakistan 2022 [2] oder die Zyklone in Mosambik 2019 [3] ist vermehrt betroffen, sondern auch der europäische Raum und damit Deutschland [4]. So kam es beispielsweise durch das Hochwasserereignis 2013 (insbesondere Elbe) zu Schäden an der Infrastruktur und zu einer hohen Belastung der Bevölkerung (z. B. durch finanzielle Einbußen [5]). Die Folgen von Hochwasserereignissen sind vielfältig und betreffen neben der Zerstörung von Wohnraum und technischer Infrastruktur vor allem auch die Gesundheit. Du et al. [6] differenzieren hinsichtlich der gesundheitlichen Folgen zwischen den sofortigen (z. B. Ertrinken, Unterkühlung, aber auch Verlust von Gesundheitspersonal durch Tod und von Gesundheitsinfrastruktur durch Zerstörung), mittelbaren (Tage bis Wochen; z. B. infizierte Wunden, Vergiftungen durch Schadstoffe im Wasser) und langfristigen Folgen (Monate bis Jahre; z. B. chronische Krankheiten, Behinderungen).

Die meisten Todesfälle bei Hochwasserereignissen erfolgen durch direktes Ertrinken. Aber auch Verletzungen bei der Flucht und Evakuierungsmaßnahmen, zu einem späteren Zeitpunkt auch mittelbar durch Aufräumarbeiten, sind von Bedeutung [6]. Weitere mittelbare Folgen umfassen Infektionskrankheiten. Ihr Risiko ist aufgrund der resilienteren Infrastruktur im globalen Norden, wie beispielweise im europäischen Raum, geringer als im globalen Süden. Ein Anstieg von Durchfallerkrankungen wurde aber auch nach Überflutungen in den USA und im Vereinigten Königreich beobachtet [7–9]. Zahlreiche Studien [10–13] konnten den Zusammenhang von Hochwasserereignissen mit deutlichen Belastungen für die psychische Gesundheit nachweisen. So zeigte sich in einer englischen Untersuchung ein mindestens 2‑fach erhöhtes Risiko für Schlafstörungen, allgemeinen psychischen Stress und Symptome einer posttraumatischen Störung [7, 14]. Langfristig bestehende hohe psychische Belastung in Form von posttraumatischen Störungen, Ängsten, Depression sowie Suizidgedanken wurden auch noch 6 Monate nach einem Hochwasserereignis in New South Wales, Australien, dokumentiert. Die psychischen Folgen sind aber nicht bei allen Betroffenen gleich und unterscheiden sich auch im Schweregrad. Nasir et al. [15] konnten feststellen, dass der Schweregrad der Belastung von der Lebenserfahrung, der Wahrnehmung der Ereignisse sowie auch Alter und Geschlecht abhängen. Weitere langfristige Folgen umfassen körperliche Behinderungen aufgrund von Verletzungen.

Das Ahrtal-Hochwasser im Juli 2021 forderte in Nordrhein-Westfalen und Rheinland-Pfalz 183 Todesopfer und über 750 Verletzte. Durch die Beschädigung von 4 Kliniken [16], über 100 Arztpraxen [17], mehr als 60 Apotheken [18] und zahlreichen Pflegeeinrichtungen wurde zudem die Gesundheitsversorgung erheblich eingeschränkt. Bislang ist unklar, welche Folgen das Hochwasserereignis für die Gesundheit der Bevölkerung hatte. In dieser Studie werden auf Grundlage von Routinedaten der Betriebskrankenkassen die raumzeitlichen Assoziationen des Ahrtal-Hochwassers mit der Gesundheit der Bevölkerung analysiert. Dabei sollen die beiden folgenden Fragen beantwortet werden:

1. Wie haben sich die ambulant und stationär gestellten Diagnosen im 3. Quartal 2021 im Vergleich zum Referenzzeitraum 3. Quartal 2020 hinsichtlich ihrer Häufigkeit in der Ahrtal-Region verändert?
2. Wie unterscheiden sich mögliche Veränderungen der Diagnosehäufigkeit von der allgemeinen Entwicklung in Deutschland?

## Material und Methoden

### Datensatz und Datenvorbereitung.

Bei dieser Studie handelt es sich um eine retrospektive Studie mit 2 Erhebungszeiträumen auf Grundlage bundesweiter stationärer und ambulanter Abrechnungsdaten des BKK-Landesverbands NORDWEST (BKK-LV NW). Insgesamt sind etwa 11,08 Mio. [19] Menschen in Deutschland in einer Betriebskrankenkasse versichert, davon haben etwa 3,08 Mio. ihren Wohnsitz im Zuständigkeitsbereich des BKK-LV NW. Der hier verwendete Datensatz beinhaltete 10,95 Mio. gesetzlich Versicherte (Stichtag: 01.07.2021). Davon lebten 132.561 in der untersuchten Region und 10,82 Mio. in der Vergleichsregion (Deutschland ohne Ahrtal).

Untersuchungszeitraum war das Quartal 3 (Q3) des Jahres 2021. Es beinhaltet den Zeitpunkt der Flutkatastrophe (14.07.2021) und einen Folgezeitraum von etwa 11 Wochen. Dieser Zeitraum wurde mit demselben Quartal des vorherigen Jahres 2020 als Referenzzeitraum verglichen. Auf eine Ausweitung des Referenzzeitraums, beispielsweise über mehrere Jahre (und Quartale), wurde aus methodischen Gründen verzichtet (z. B. Ungenauigkeiten bei der Verwendung eventueller Durchschnittswerte, Einfluss der Coronapandemie beim Inanspruchnahmeverhalten). Die Daten lagen in Form der Anzahl abgerechneter Fälle mit der jeweiligen ICD-10-Diagnose vor. Aus Datenschutzgründen war allerdings keine Untersuchung auf Ebene der Wohnortanschriften möglich, sodass die Fallzahlen auf Grundlage der medizinischen Betriebsstätten (hier nach § 295 SGB V abrechnende Arztpraxen) betrachtet wurden.

### Untersuchungsregion

**Figure 1:**
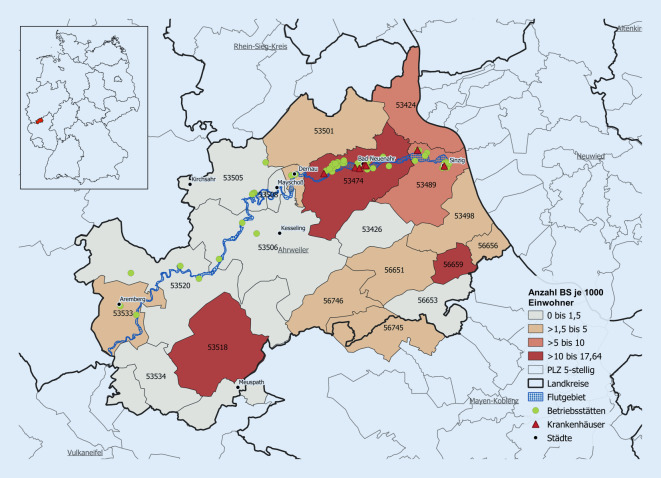

Als Untersuchungsregion wurde hier die 2‑stellige Postleitzahl-Region 53 gewählt. Diese Region umfasst mehrere Städte und Landkreise in den Bundesländern Rheinland-Pfalz und Nordrhein-Westfalen. Innerhalb dieser Region befindet sich auch die Kernregion der Flutkatastrophe entlang der Ahr mit den Postleitzahlgebieten 53533, 53520, 53506, 53505, 53508, 53507, 53474, und 53489 im Kreis Ahrweiler. Die Untersuchungsregion wurde so gewählt, dass umliegende Landkreise miteingeschlossen sind, um den Effekt der Mitversorgung von Patienten aus der Kernregion miterfassen zu können. Darüber hinaus konnte so eine größere Fallzahl erreicht werden. In Abb. 1 sind neben dem primären Hochwassergebiet zusätzlich Einrichtungen der ambulanten Versorgung (Betriebsstätten) und stationären Versorgung ersichtlich.

### Analysen

Die Diagnosen (nach ICD-10) wurden für ambulante Fälle, stationäre Fälle sowie ambulante und stationäre Fälle in der Schwangerschaft getrennt untersucht. Nach einer deskriptiven Betrachtung wurden alle Fallzahlen der Untersuchungsregion von übermittelten ICD-10-Diagnosen durch Berechnung von Veränderungsraten zwischen Q3 in 2020 und 2021 auf Unterschiede überprüft. Für die hierbei auffälligen Diagnosen wurden Prävalenzraten gebildet. Als Grundgesamtheit galten die jeweiligen Gesamtfallzahlen an stationären oder ambulanten Diagnosen für das jeweilige Quartal. Die Veränderung der Prävalenzraten zwischen beiden Zeiträumen wurde daraufhin mit einem Rate-Ratio-Test (Prevalence Rate Ratio = PRR) auf Signifikanz geprüft. Um zu untersuchen, inwieweit diese Veränderungen in einem Zusammenhang mit dem Hochwasserereignis im Ahrtal stehen und nicht eine allgemeine Tendenz darstellen, wurden die Ergebnisse mit dem Rest Deutschlands (Gesamtdeutschland ohne Ahrtal) verglichen. Dabei wurde überprüft, ob sich signifikante PRRs der Untersuchungsregion in vergleichbarer Weise (Zunahme oder Abnahme) auch in der Vergleichsregion zeigten. Bei den Analysen wurde ein *p*-Wert von < 0,05 als statistisch signifikant angesehen. Zur Visualisierung wurden die Zu- und Abnahmen der einzelnen Diagnosegruppen zwischen Q3 2020 und Q3 2021 in Form eines Venn-Diagramms dargestellt. Die Größe der Kästchen im Diagramm ist flächenproportional zur Häufigkeit der jeweiligen Diagnosegruppe.

Die Daten wurden in Excel (Microsoft, Redmond, USA, 2022) aufbereitet und mit R 4.1.2 (R Core Team, Wien, Österreich 2021), Package „rateratio.test“ [20] analysiert. Darüber hinaus wurde Q‑GIS 3.22.13-Białowieża angewendet.

## Ergebnisse

Zwischen Q3 2020 und Q3 2021 stieg die Anzahl aller im niedergelassenen Bereich mit Wohnort in der Untersuchungsregion erfassten Patienten von 99.139 auf 104.639 leicht an. In der Vergleichsregion betrug die Zahl der Patienten 7,99 Mio. (2020). bzw. 8,41 Mio. (2021). In Hinblick auf den stationären Sektor stieg die Anzahl der Krankenhausfälle im übrigen Deutschland von 1013 auf 1034 Mio. Fälle leicht an. In den Krankenhäusern der Untersuchungsregion ging die Fallzahl dagegen zurück: von 15.046 in Q3 2020 auf 14.664 in Q3 2021. Im Ahrtal wurden in Q3 2021 für den ambulanten Sektor 8391 verschiedene (gesicherte) Diagnosen 961.281-mal abgerechnet (Q3 2020: 8476 Diagnosen 966.984-mal).

### Stationäre Diagnosen

Für den stationären Bereich wurden in der Untersuchungsregion Diagnosen identifiziert, die in Hinblick auf Zu- und Abnahmen auffällig waren. Zugenommen haben zwischen Q3 2020 und Q3 2021 u. a.: *depressive Episoden* (ICD-10 F32; PRR 0,86), *Unwohlsein und Ermüdung* (ICD-10 R53; PRR 0,61) und *spezielle Verfahren zur Untersuchung auf infektiöse und parasitäre Krankheiten* (ICD-10 Z11, PRR 0,67). Diagnosen mit einer Abnahme zwischen den Quartalen umfassten beispielsweise die *unerwünschten Nebenwirkungen* (ICD-10 T78; PRR 2,36) und die *Nichtrheumatischen Trikuspidalklappenkrankheiten *(ICD-10 I36; PRR 1,66).

**Figure 2:**
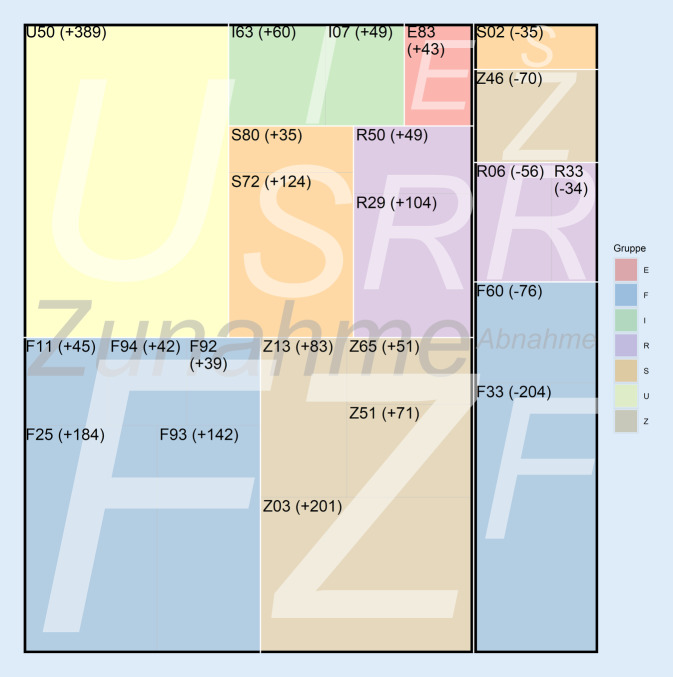

Für den stationären Bereich wurden 17 Diagnosen identifiziert, die im Ahrtal zugenommen und im übrigen Deutschland abgenommen haben bzw. unverändert geblieben sind (Tab. 1). Der Vergleich zeigt, dass im Ahrtal 5 psychische Diagnosen teilweise deutlich zugenommen haben (PRR 0,73–0,30), wie z. B. *Emotionale Störungen des Kindesalters *(ICD-10 F93; PRR 0,40). Daneben waren etwa *Frakturen des Femurs* (ICD-10 S72; PRR 0,64) und auch *Fieber sonstiger und unbekannter Ursache* (ICD-10 R50; PRR 0,74) auffällig häufiger. Gegenteiliges, d. h. eine leichte Abnahme im Ahrtal (und Zunahme bzw. Nicht-Veränderung in Deutschland), wurde bei 6 ICD-Kodes, beispielsweise bei 2 psychischen Diagnosen, den *spezifischen Persönlichkeitsstörungen* (ICD-10 F60; PRR 1,31) und den *rezidivierenden depressiven Störungen* (F33; PRR 1,16), gefunden (Tab. 1 und Abb. 2).

**Table 1:** 

	Diagnosegruppe	ICD-10	Bezeichnung ICD	Ahrtal PLZ 53−				Deutschland ohne Ahrtal PLZ 53−			
				Q3 2020	Q3 2021	Prevalence Rate Ratio	*p*-Wert	Q3 2020	Q3 2021	Prevalence Rate Ratio	*p*-Wert
Zunahme	E	E83	Störungen des Mineralstoffwechsels	96	139	0,73	0,0167	6923	6841	1,01	0,5397
	F	F11	Psychische und Verhaltensstörungen durch Opioide	101	146	0,73	0,0146	5873	5617	1,04	0,0217
		F25	Schizoaffektive Störungen	109	293	0,39	< 0,0001	9149	9025	1,01	0,4101
		F92	Kombinierte Störung des Sozialverhaltens und der Emotionen	16	55	0,31	< 0,0001	5531	4653	1,19	< 0,0001
		F93	Emotionale Störungen des Kindesalters	87	229	0,40	< 0,0001	11.163	11.010	1,01	0,3561
		F94	Störungen sozialer Funktionen mit Beginn in der Kindheit und Jugend	40	82	0,51	0,0004	2953	2805	1,05	0,0591
	I	I07	Rheumatische Trikuspidalklappenkrankheiten	11	60	0,19	< 0,0001	1501	1438	1,04	0,2679
		I63	Hirninfarkt	238	298	0,84	0,0491	24.370	24.743	0,98	0,0681
	R	R29	Sonstige Symptome, Nervensystem und Muskel-Skelett-System betreffend	200	304	0,69	0,0001	18.829	19.161	0,98	0,0678
		R50	Fieber sonstiger und unbekannter Ursache	116	165	0,74	0,0134	11.795	10.758	1,09	< 0,0001
	S	S72	Fraktur des Femurs	193	317	0,64	< 0,0001	18.766	18.651	1,01	0,6439
		S80	Oberflächliche Verletzung des Unterschenkels	79	114	0,73	0,0319	6810	6527	0,99	0,0180
	U	U50	Motorische Funktionseinschränkung	765	1154	0,69	< 0,0001	77.334	77.910	0,99	0,0857
		Z03	Ärztliche Beobachtung und Beurteilung von Verdachtsfällen, Verdacht ausgeschlossen	691	892	0,82	0,0001	56.151	53.924	1,04	< 0,0001
	Z	Z13	Spezielle Verfahren zur Untersuchung auf sonstige Krankheiten oder Störungen	242	325	0,79	0,0044	8022	7501	1,07	< 0,0001
		Z51	Sonstige medizinische Behandlung	100	171	0,60	0,0001	14.897	14.944	0,99	0,7041
		Z65	Kontaktanlässe mit Bezug auf andere psychosoziale Umstände	1	52	0,02	< 0,0001	187	224	0,83	0,0735
Abnahme	F	F33	Rezidivierende depressive Störung	2230	2026	1,16	< 0,0001	118.760	139.267	0,85	< 0,0001
		F60	Spezifische Persönlichkeitsstörungen	382	306	1,32	0,0003	22.225	23.373	0,95	< 0,0001
	R	R06	Störungen der Atmung	487	431	1,19	0,0077	30.917	31.979	0,97	< 0,0001
		R33	Harnverhaltung	99	65	1,61	0,0027	6334	6463	0,98	0,2279
	S	S02	Fraktur des Schädels und der Gesichtsschädelknochen	126	91	1,46	0,0056	9414	9620	0,98	0,1149
	Z	Z46	Versorgen mit und Anpassen von anderen medizinischen Geräten oder Hilfsmitteln	250	180	1,47	0,0001	14.178	14.476	0,98	0,0621

ICD-10: Internationale statistische Klassifikation der Krankheiten und verwandter Gesundheitsprobleme, Version 10

Bei alleiniger Betrachtung des Untersuchungszeitraumes wurden Auffälligkeiten im Kontext von Schwangerschaft und Geburt ersichtlich. Zum Beispiel zeigte sich eine Zunahme von *übertragenen Schwangerschaften *(ICD-10 O48; PRR 0,58). Im Vergleich mit dem restlichen Deutschland konnte im Ahrtal eine Abnahme der Diagnose *Spontangeburt eines Einlings* (ICD-10 O80; PRR 1,30) festgestellt werden. Die *Geburt eines Einlings durch Schnittentbindung* (ICD-10 O82; PRR 0,71) hat sowohl im Ahrtal als auch bundesweit zugenommen. Damit stieg die Rate für Schnittentbindungen von 23,2 % im Q3 2020 auf 35,4 % in Q3 2021, im Rest Deutschlands von 16,8 % auf 17,4 %.

### Ambulante Diagnosen

Bei einigen ambulanten Diagnosen im Untersuchungszeitraum zeigte sich zwischen den Quartalen eine Zunahme. Dazu zählen unter anderem *Reaktionen auf schwere Belastungen und Anpassungsstörungen* (ICD-10 F43; PRR 0,94), *sonstige Komplikationen bei chirurgischen Eingriffen und medizinischen Behandlungen* (ICD-10 T88; PRR 0,81) oder auch, wie schon im stationären Sektor, *spezielle Verfahren zur Untersuchung auf infektiöse und parasitäre Krankheiten* (ICD-10 Z11; PRR 0,72). Eine Abnahme zeigte sich beispielsweise bei den Z‑Diagnosen wie *speziellen Verfahren zur Untersuchung auf Neubildungen* (ICD-10 Z12; PRR 1,21) oder *sonstigen medizinischen Behandlungen* (ICD-10 Z51; PRR 1,18).

**Figure 3:**
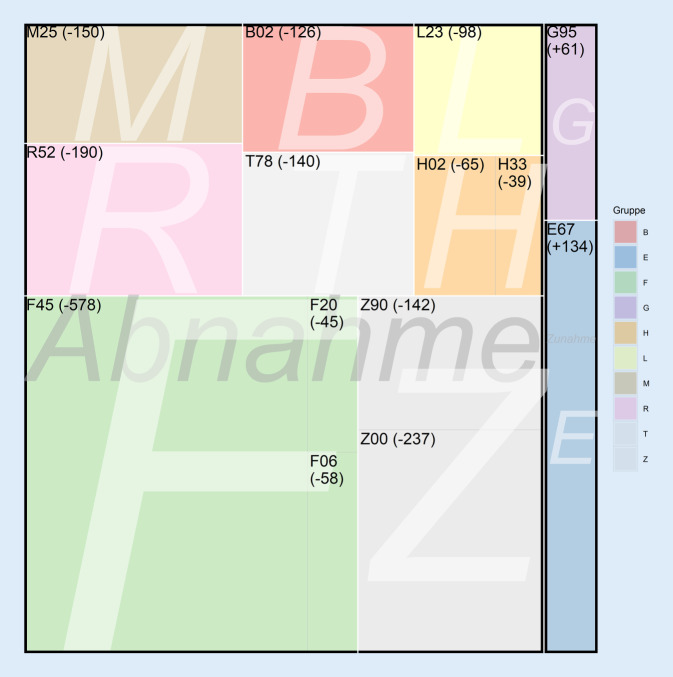

Für das Ahrtal wurde eine deutliche Zunahme der Diagnose ICD-10 E67 (*sonstige Überernährung*; PRR 0,25) beobachtet, parallel für das übrige Deutschland eine Abnahme. Einige Diagnosen zeigten im Ahrtal eine Abnahme und gleichzeitig im übrigen Deutschland eine Zunahme, z. B. die *Somatoformen Störungen* (ICD-10 F45; PRR 1,14), und wenige weitere Diagnosen wie ICD-10 B02, *Herpes Zoster *(PRR 1,35), und verschiedene unspezifische Diagnoseschlüssel wie *Allgemeinuntersuchung und Abklärung ohne Beschwerden oder angegebene Diagnose* (ICD-10 Z00; PRR 1,09) oder auch *unerwünschte Nebenwirkungen, andernorts nicht klassifiziert* (ICD-10 T78; PRR 1,08; Tab. 2 und Abb. 3). Im Kontext der Schwangerschaften zeigten sich im Ahrtal im Vergleich zur restlichen Bundesrepublik (ambulant) keine Auffälligkeiten.

**Table 2:** 

	Diagnosegruppe	ICD-10	Bezeichnung ICD	Ahrtal PLZ 53−				Deutschland ohne Ahrtal PLZ 53−			
				Q3 2020	Q3 2021	Prevalence Rate Ratio	*p*-Wert	Q3 2020	Q3 2021	Prevalence Rate Ratio	*p*-Wert
Zunahme	E	E67	Sonstige Überernährung	41	175	0,25	< 0,0001	5248	5285	0,99	0,6758
	F	G95	Sonstige Krankheiten des Rückenmarks	158	219	0,76	0,0088	11.360	11.266	1,01	0,6035
Abnahme	B	B02	Zoster (Herpes zoster)	578	452	1,35	< 0,0001	39.101	39.295	0,99	0,3825
	F	F06	Andere psych. Störungen aufgrund einer Schädigung oder Funktionsstörung des Gehirns	730	672	1,15	0,0105	59.893	59.177	1,01	0,0650
		F20	Schizophrenie	601	556	1,14	0,0251	38.991	38.550	1,01	0,1624
		F45	Somatoforme Störungen	7668	7090	1,14	< 0,0001	667.831	670.797	0,99	< 0,0001
	H	H02	Sonstige Affektionen des Augenlides	771	706	1,15	0,0064	50.514	51.502	0,98	< 0,0001
		H33	Netzhautablösung und Netzhautriss	325	286	1,20	0,0248	22.694	22.775	0,99	0,6063
	L	L23	Allergische Kontaktdermatitis	724	626	1,2	0,0003	63.698	63.370	1,00	0,4946
	M	M25	Sonstige Gelenkkrankheiten, anderenorts nicht klassifiziert	5143	4993	1,09	< 0,0001	364.833	376.038	0,97	< 0,0001
	R	R52	Schmerz, anderenorts nicht klassifiziert	5940	5750	1,09	< 0,0001	481.981	488.035	0,99	< 0,0001
	T	T78	Unerwünschte Nebenwirkungen, anderenorts nicht klassifiziert	5318	5178	1,08	< 0,0001	344.988	353.567	0,97	< 0,0001
	Z	Z00	Allgemeinuntersuchung und Abklärung ohne Beschwerden oder angegebene Diagnose	6380	6143	1,10	< 0,0001	517.141	549.713	0,94	< 0,0001
		Z90	Verlust von Organen, anderenorts nicht klassifiziert	2183	2041	1,13	0,0001	155.498	157.295	0,99	0,0003

ICD-10: Internationale statistische Klassifikation der Krankheiten und verwandter Gesundheitsprobleme, Version 10

## Diskussion

Diese Studie hatte zum Ziel, erstmals die räumlichen und zeitlichen Assoziationen der Ahrtal-Hochwasserkatastrophe im Jahr 2021 mit der Gesundheit der lokalen Bevölkerung anhand von Krankenkassendaten für das Q3 2020 und 2021 zu untersuchen. Die Daten zeigen im zeitlichen Zusammenhang mit dem Ahrtal-Hochwasser eine deutliche Zunahme der Kodierungen in einigen Diagnosegruppen. Im stationären Bereich wurde eine Zunahme innerhalb der F‑Diagnosen (*psychische und Verhaltensstörungen*) verzeichnet. Daneben waren vor allem S‑Diagnosen (*Verletzungen*) und verschiedene Diagnoseschlüssel innerhalb der Z‑Kodierungen (*Faktoren, die den Gesundheitszustand beeinflussen und zur Inanspruchnahme des Gesundheitswesens führen*) hinsichtlich einer Zunahme auffallend. Zudem ließen sich noch Zunahmen von Einzeldiagnosen der Gruppen R (*Symptome und abnorme klinische und Laborbefunde, die anderenorts nicht klassifiziert sind), *M (*Krankheiten des Muskel-Skelett-Systems und des Bindegewebes*), B (*bestimmte infektiöse und parasitäre Krankheiten*) und schwangerschaftsassoziierten Diagnosen nachweisen. Im ambulanten Sektor zeigte sich überwiegend eine leichte Abnahme verschiedener Diagnosen.

Die erhöhte Inanspruchnahme des stationären Sektors betrifft zum einen somatische Akutdiagnosen wie Femurfrakturen, Oberschenkelverletzungen und die Notwendigkeit einer ärztlichen Beobachtung nach Ausschluss einer Verdachtsdiagnose. Dies sind typische und direkte gesundheitliche Folgen von Hochwasserereignissen, wie es beispielsweise auch aus den Studien von Du et al. [6] und Ahern et al. [21] hervorgeht. Zum anderen sind vielseitige psychische Belastungsreaktionen wie emotionale Störungen bei Kindern, schizoaffektive Störungen und Missbrauch von Opioiden auffallend. Diese Ergebnisse passen zu Berichten, die einen erheblichen psychologischen und psychiatrischen Versorgungsbedarf bei von der Flut Betroffenen zeigen [22], etwa jener vom ad hoc eingerichteten Traumahilfezentrum Ahrtal [23]. Neben den somatischen Akutdiagnosen zählen die psychischen Belastungen zu den besonders häufigen Folgen von Hochwasserereignissen (z. B. Asim et al. [24], Matthews et al. [25], Aldermann et al. [10], Fewtrell und Kay [26]). Zusätzlich könnte auch die gesteigerte Rate von Schnittentbindungen eine Folge der psychischen Belastung in der Region sein, worauf beispielsweise die Studie von Ko [27] hindeutet. Zu langfristigen psychischen Folgen liefert die vorliegende Studie aufgrund des begrenzten Untersuchungszeitraums keine Daten. In Bezug auf beispielsweise die posttraumatische Belastungsstörung (PTBS) werden jedoch 4000 Betroffene in der Ahrtal-Region geschätzt, mit erheblichen Versorgungsengpässen [28].

Den zugenommenen Diagnosen insbesondere im stationären Bereich stehen auch abnehmende Prävalenzen gegenüber, insbesondere im ambulanten Bereich. Beispielsweise wurden weniger Zoster-Erkrankungen, aber auch verschiedene Z‑Diagnosen (z. B. ICD-10 Z00, *Allgemeinuntersuchung und Abklärung ohne Beschwerden o. angegebene Diagnose*) kodiert. Daneben zeigte sich eine leichte Zunahme übertragener Schwangerschaften. Möglicherweise hat die Hochwasserkatastrophe in diesen Fällen dazu geführt, dass der Gang zum Arzt verschoben oder komplett abgesagt und entsprechend keine Leistung in Anspruch genommen wurde. Die Hypothese, dass die medizinische Vorstellung bei leichteren Erkrankungen und Routineuntersuchungen verschoben wurde und schwerwiegendere Fälle zugenommen haben, wird damit unterstützt. Dazu kommt, dass die ambulante Versorgung zum einen durch direkte Schäden an Praxen und medizinischen Versorgungszentren, zum anderen durch logistische Schwierigkeiten, etwa durch Beschädigung von Straßen sowie privaten und öffentlichen Verkehrsmitteln, beeinträchtigt war. Dadurch war das Aufsuchen der ambulanten Versorgung mit höheren Hürden verbunden.

Die direkte Vergleichbarkeit der vorliegenden Ergebnisse, insbesondere der Diagnosehäufigkeiten, mit anderen Studien zu gesundheitlichen Folgen von Hochwasserereignissen ist insgesamt eingeschränkt, da andere Studien oftmals auf Literaturrecherchen (z. B. [6, 26]) oder Primärdatenerhebungen [10, 25] basieren. Es zeigt sich aber, dass die gesundheitlichen Folgen des Ahrtal-Hochwassers mit denen anderer geographisch vergleichbarer Ereignisse in vielen Punkten übereinstimmen und vor allem die Folgen auf die mentale Gesundheit von Bedeutung sind. Eine Zunahme von Infektionskrankheiten konnte im Ahrtal, zumindest auf Grundlage der hier verwendeten Daten, nicht identifiziert werden.

Die vorliegende Studie unterliegt Limitationen. Es ist davon auszugehen, dass die Auswirkungen der Flutkatastrophe in dieser Studie unterschätzt werden, da die Untersuchungsregion aus Datenschutzgründen (Berücksichtigung ausreichend hoher Fallzahlen) und aufgrund der Mitversorgung durch die umliegenden Regionen sehr weit gefasst werden musste und somit auch nicht direkt betroffene Regionen miteinschließt. Die alleinige Betrachtung der Veränderung von Diagnosehäufigkeiten kann Fehlinterpretationen im Kontext des Hochwasserereignisses nicht vollends ausschließen. Darüber hinaus können keine Aussagen darüber gemacht werden, ob es zu einer Leistungsverlagerung zwischen den Sektoren gekommen ist oder ob außerhalb der Hochwasserregion vermehrt Leistungen in Anspruch genommen wurden. Ferner kann die Studie keine Aussagen darüber treffen, ob bestimmte vulnerable Gruppen, etwa hinsichtlich Vorerkrankungen, Alter und Geschlechtszugehörigkeit, besonders schwer von den gesundheitlichen Auswirkungen des Hochwassers betroffen waren. In diesem Zusammenhang haben Studien insbesondere zu Hochwasser im globalen Süden eine erhöhte Betroffenheit etwa von Frauen und Kindern gezeigt (z. B. [29, 30]). Auch kann eine Stichprobenverzerrung (Selection Bias) bei den GK-Versicherten nicht vollends ausgeschlossen werden. Die Stärke dieser Studie liegt in den zugrunde liegenden Daten. Diese ermöglichen eine umfangreiche Betrachtung der gesundheitlichen Auswirkungen der Ahrtal-Hochwasserkatastrophe und lassen Rückschlüsse auf die Veränderung einzelner Diagnosen bzw. Diagnosegruppen unter Berücksichtigung eines Vergleichszeitraumes und bundesweiter Vergleichsdaten zu. Weitere Studien sollten sowohl die somatischen als auch die psychischen langfristigen Folgen des Hochwassers untersuchen. Zudem sollten die gesundheitsökonomischen Folgen genauer untersucht werden.

Der Klimawandel erhöht die Wahrscheinlichkeit bisher nicht eingetretener und damit schwer antizipierbarer Extremwetterereignisse. Diese Untersuchung zeigt vor allem im stationären Bereich eine Erhöhung der Krankheitslast im direkten zeitlichen Zusammenhang mit dem Hochwasser im Ahrtal. Gleichzeitig wurden viele Gesundheitseinrichtungen durch das Hochwasser so getroffen, dass sie keine Versorgung mehr leisten konnten [31]. Es ist davon auszugehen, dass dies insbesondere den ambulanten Sektor betroffen hat, was auch die hier identifizierte Zunahme von Diagnosen im stationären Sektor stützt.

## Fazit

Dieser Artikel ist in Deutschland unserem Wissen nach der Erste seiner Art. Insofern kann er nur als „Ausgangspunkt“ für das Thema angesehen werden. In Planung ist eine gesundheitsökonomische Bewertung des Ahrtal-Hochwassers, um die aus solch einem Ereignis entstehenden Kosten, u. a. für das Gesundheitssystem, beziffern zu können. Grundsätzlich wäre es wünschenswert, wenn weitere Untersuchungen zu anderen Hochwasserkatastrophen (z. B. Elbe-Hochwasser 2013) und deren Konsequenzen für die Gesundheit folgen würden. Somit könnten Vergleiche vorgenommen werden, deren Erkenntnisse wiederum in Maßnahmen zur Reduzierung der gesundheitlichen Auswirkungen solcher Ereignisse münden. Die Ergebnisse dieser Studie verdeutlichen eine weitere Facette, auf welche Weise die Klimakrise die Krankheitslast in Deutschland zukünftig erhöhen wird.
